# Risk Factors for Hospitalization, Mechanical Ventilation, or Death Among 10 131 US Veterans With SARS-CoV-2 Infection

**DOI:** 10.1001/jamanetworkopen.2020.22310

**Published:** 2020-09-23

**Authors:** George N. Ioannou, Emily Locke, Pamela Green, Kristin Berry, Ann M. O’Hare, Javeed A. Shah, Kristina Crothers, McKenna C. Eastment, Jason A. Dominitz, Vincent S. Fan

**Affiliations:** 1 Division of Gastroenterology, Veterans Affairs Puget Sound Healthcare System and University of Washington, Seattle; 2 Research and Development, Veterans Affairs Puget Sound Health Care System, Seattle, Washington; 3 Division of Nephrology, Veterans Affairs Puget Sound Healthcare System and University of Washington, Seattle; 4 Division of Allergy and Infectious Disease, Veterans Affairs Puget Sound Healthcare System and University of Washington, Seattle; 5 Division of Pulmonary and Critical Care, Veterans Affairs Puget Sound Healthcare System and University of Washington, Seattle

## Abstract

**Importance:**

Identifying independent risk factors for adverse outcomes in patients infected with severe acute respiratory syndrome coronavirus 2 (SARS-CoV-2) can support prognostication, resource utilization, and treatment.

**Objective:**

To identify excess risk and risk factors associated with hospitalization, mechanical ventilation, and mortality in patients with SARS-CoV-2 infection.

**Design, Setting, and Participants:**

This longitudinal cohort study included 88 747 patients tested for SARS-CoV-2 nucleic acid by polymerase chain reaction between Feburary 28 and May 14, 2020, and followed up through June 22, 2020, in the Department of Veterans Affairs (VA) national health care system, including 10 131 patients (11.4%) who tested positive.

**Exposures:**

Sociodemographic characteristics, comorbid conditions, symptoms, and laboratory test results.

**Main Outcomes and Measures:**

Risk of hospitalization, mechanical ventilation, and death were estimated in time-to-event analyses using Cox proportional hazards models.

**Results:**

The 10 131 veterans with SARS-CoV-2 were predominantly male (9221 [91.0%]), with diverse race/ethnicity (5022 [49.6%] White, 4215 [41.6%] Black, and 944 [9.3%] Hispanic) and a mean (SD) age of 63.6 (16.2) years. Compared with patients who tested negative for SARS-CoV-2, those who tested positive had higher rates of 30-day hospitalization (30.4% vs 29.3%; adjusted hazard ratio [aHR], 1.13; 95% CI, 1.08-1.13), mechanical ventilation (6.7% vs 1.7%; aHR, 4.15; 95% CI, 3.74-4.61), and death (10.8% vs 2.4%; aHR, 4.44; 95% CI, 4.07-4.83). Among patients who tested positive for SARS-CoV-2, characteristics significantly associated with mortality included older age (eg, ≥80 years vs <50 years: aHR, 60.80; 95% CI, 29.67-124.61), high regional COVID-19 disease burden (eg, ≥700 vs <130 deaths per 1 million residents: aHR, 1.21; 95% CI, 1.02-1.45), higher Charlson comorbidity index score (eg, ≥5 vs 0: aHR, 1.93; 95% CI, 1.54-2.42), fever (aHR, 1.51; 95% CI, 1.32-1.72), dyspnea (aHR, 1.78; 95% CI, 1.53-2.07), and abnormalities in the certain blood tests, which exhibited dose-response associations with mortality, including aspartate aminotransferase (>89 U/L vs ≤25 U/L: aHR, 1.86; 95% CI, 1.35-2.57), creatinine (>3.80 mg/dL vs 0.98 mg/dL: aHR, 3.79; 95% CI, 2.62-5.48), and neutrophil to lymphocyte ratio (>12.70 vs ≤2.71: aHR, 2.88; 95% CI, 2.12-3.91). With the exception of geographic region, the same covariates were independently associated with mechanical ventilation along with Black race (aHR, 1.52; 95% CI, 1.25-1.85), male sex (aHR, 2.07; 95% CI, 1.30-3.32), diabetes (aHR, 1.40; 95% CI, 1.18-1.67), and hypertension (aHR, 1.30; 95% CI, 1.03-1.64). Notable characteristics that were not significantly associated with mortality in adjusted analyses included obesity (body mass index ≥35 vs 18.5-24.9: aHR, 0.97; 95% CI, 0.77-1.21), Black race (aHR, 1.04; 95% CI, 0.88-1.21), Hispanic ethnicity (aHR, 1.03; 95% CI, 0.79-1.35), chronic obstructive pulmonary disease (aHR, 1.02; 95% CI, 0.88-1.19), hypertension (aHR, 0.95; 95% CI, 0.81-1.12), and smoking (eg, current vs never: aHR, 0.87; 95% CI, 0.67-1.13). Most deaths in this cohort occurred in patients with age of 50 years or older (63.4%), male sex (12.3%), and Charlson Comorbidity Index score of at least 1 (11.1%).

**Conclusions and Relevance:**

In this national cohort of VA patients, most SARS-CoV-2 deaths were associated with older age, male sex, and comorbidity burden. Many factors previously reported to be associated with mortality in smaller studies were not confirmed, such as obesity, Black race, Hispanic ethnicity, chronic obstructive pulmonary disease, hypertension, and smoking.

## Key Points

**Question:**

What are the risk factors associated with hospitalization, mechanical ventilation, and death among patients with severe acute respiratory syndrome coronavirus 2 (SARS-CoV-2) infection?

**Findings:**

In this national cohort study of 88 747 veterans tested for SARS-CoV-2, hospitalization, mechanical ventilation, and mortality were significantly higher in patients with positive SARS-CoV-2 test results than among those with negative test results. Significant risk factors for mortality included older age, high regional coronavirus disease 2019 burden, higher Charlson Comorbidity Index score, fever, dyspnea, and abnormal results in many routine laboratory tests; however, obesity, Black race, Hispanic ethnicity, chronic obstructive pulmonary disease, hypertension, and smoking were not associated with mortality.

**Meaning:**

In this study, most deaths from SARS-CoV-2 occurred in patients with age of 50 years or older, male sex, and greater comorbidity burden.

## Teaser

This cohort study identifies excess risk and risk factors associated with hospitalization, mechanical ventilation, and mortality in patients with severe acute respiratory syndrome coronavirus 2 (SARS-CoV-2).

## Introduction

Infection with severe acute respiratory syndrome coronavirus 2 (SARS-CoV-2) has a very broad spectrum of clinical severity, ranging from asymptomatic infection to life-threatening illness.^1,2^ It remains unclear why some patients infected with SARS-CoV-2 develop the severe complications of coronavirus disease 2019 (COVID-19), which include acute respiratory distress syndrome (ARDS) and death.

Multiple risk factors for developing severe COVID-19 disease have been reported, including sociodemographic factors and comorbid conditions.^1,3,4,5,6,7,8,9,10,11,12,13,14,15,16,17,18,19,20,21,22,23,24^ However, most prior studies, particularly those published earlier in the course of the pandemic, did not include multivariable adjustment to identify independent risk factors, and few studies examined a range of different disease outcomes, including hospitalization, mechanical ventilation, and death. Most prior studies have been local or regional, rather than national, in scope. Finally, most studies have not compared patients who tested positive for SARS-CoV-2 with those who tested negative to determine the excess risk associated with SARS-CoV-2 infection itself as opposed to other underlying comorbid conditions in patients who happen to have SARS-CoV-2 infection. To address this knowledge gap, we used national data from the Department of Veterans Affairs (VA) health care system to determine the risk of hospitalization, mechanical ventilation, and death associated with infection and to identify characteristics independently associated with these outcomes in patients who tested positive for SARS-CoV-2.

## Methods

### Data Source and Study Population

The VA supports the largest integrated national health care system in the United States and provides care for more than 6 million veterans annually. The VA uses a single, national comprehensive electronic health care information network. We derived data from the VA’s Corporate Data Warehouse, a data repository of electronic medical records, developed by the VA Informatics and Computing Infrastructure (VINCI) to facilitate research. To support COVID-19 research, VINCI analysts created and are continually updating the COVID-19 Shared Data Resource,^25^ which includes analytic variables extracted from the Corporate Data Warehouse for all VA enrollees tested for SARS-CoV-2. Using this resource, we identified all VA enrollees (N = 88 747) who had nasopharyngeal swabs tested for SARS-CoV-2 nucleic acid by polymerase chain reaction in inpatient or outpatient VA facilities (including VA nursing homes) between February 28 and May 14, 2020, for any indication, excluding those who were VA employees. Most tests were performed in VA laboratories using the US Food and Drug Administration–approved RealTime (Abbott Laboratories) or Xpert-Xpress (Cepheid) SARS-CoV-2 assays; some were sent to commercial or state public health laboratories, especially during the early days of the pandemic. Cohort members were followed up through June 22, 2020, for study outcomes allowing for a minimum follow-up of 39 days. This study was approved by the institutional review board of the Veterans Affairs Puget Sound Healthcare System, which granted a waiver of informed consent because this was a retrospective cohort study based on an existing database. This study followed the Strengthening the Reporting of Observational Studies in Epidemiology (STROBE) reporting guideline.

### Definition of Positive or Negative SARS-CoV-2 Status and Index Date

Patients were defined as positive for SARS-CoV-2 if they had at least 1 positive polymerase chain reaction test (n = 10 131 [11.4%]) during the ascertainment period. Patients were defined as negative for SARS-CoV-2 if all polymerase chain reaction tests were negative (n = 78 616 [88.6%]). Final adjudication of SARS-CoV-2 status was performed by the VA National Surveillance Tool, which is intended to be the single, authoritative data source for the determination of positive and negative cases within the Veterans Health Administration. The *index date* for all analyses was defined as the date of the earliest positive test (for patients with SARS-CoV-2) or the date of the earliest negative test (for patients with no SARS-CoV-2), unless the patient had been admitted to a VA hospital during the preceding 15 days, in which case the date of admission served as the index date.

### Adverse Outcomes

We determined the following 3 outcomes: (1) hospitalization at the index date or within 15 days of the index date, (2) mechanical ventilation at the index date or within 60 days, and (3) all-cause mortality at any time after the index date. Deaths that occurred both in and out of the VA are comprehensively captured in the Corporate Data Warehouse through a variety of sources, including VA inpatient files, VA Beneficiary Identification and Records Locator System, Social Security Administration death files, and the Department of Defense.^26^ Episodes of mechanical ventilation and hospitalization that occurred outside the VA were captured only if the VA paid for these hospitalizations at non-VA facilities under the VA Community Care program.

### Baseline Characteristics Evaluated for Associations With Adverse Outcomes

We included characteristics that were evaluated in prior studies or that we hypothesized might be associated with adverse outcomes. Baseline sociodemographic characteristics included age, sex, race, ethnicity, body mass index (calculated as weight in kilograms divided by height in meters squared), urban vs rural residence (based on zip codes), and regional per capita COVID-19–related mortality, operationalized as the number of COVID-19–related deaths per million residents in each participant’s state of residence as of June 11, 2020 (Table 1).^27^

**Table 1.** Associations Between Sociodemographic Characteristics and Hospitalization, Mechanical Ventilation, or Mortality Among 10 131 Patients Who Tested Positive for SARS-CoV-2 Between February 28 and May 14, 2020

Demographic factor	Patients, No. (%)	Hospitalization			Mechanical ventilation				Mortality			
		30-d Rate, %	Hazard ratio (95% CI)		30-d Rate, %	Hazard ratio (95% CI)			30-d Rate, %	Hazard ratio (95% CI)		
			Age-adjusted	Adjusted^a^		Age-adjusted		Adjusted^a^		Age-adjusted		Adjusted^a^
All patients	10 131 (100)	34.2	NA	NA	6.7	NA	NA		10.8	NA	NA	
Sex												
Women	910 (9.0)	19.6	1 [Reference]	1 [Reference]	2.1	1 [Reference]	1 [Reference]		2.8	1 [Reference]	1 [Reference]	
Men	9221 (91.0)	35.7	1.37 (1.18-1.60)	1.22 (1.04-1.42)	7.2	2.28 (1.43-3.63)	2.07 (1.30-3.32)		11.6	1.52 (1.02-2.25)	1.38 (0.93-2.06)	
Age, y			1.03 (1.02-1.03)	1.02 (1.02-1.03)		1.03 (1.02-1.03)	1.03 (1.02-1.03)			1.07 (1.07-1.08)	1.07 (1.06-1.08)	
18-49	1973 (19.5)	14.9	1 [Reference]	1 [Reference]	1.6	1 [Reference]	1 [Reference]		0.4	1 [Reference]	1 [Reference]	
50-64	2917 (28.8)	30.7	2.21 (1.94-2.52)	1.76 (1.53-2.02)	6.0	3.89 (2.65-5.70)	2.72 (1.82-4.05)		4.1	10.35 (5.06-21.19)	9.27 (4.51-19.08)	
65-79	3724 (36.8)	43.4	3.27 (2.88-3.70)	2.40 (2.08-2.77)	10.1	6.69 (4.63-9.67)	4.32 (2.88-6.47)		13.8	36.37 (18.08-73.16)	27.47 (13.48-55.99)	
≥80	1517 (15.0)	44.1	3.62 (3.15-4.16)	2.94 (2.50-3.46)	6.7	4.87 (3.24-7.31)	3.98 (2.54-6.24)		29.7	82.22 (40.82-165.63)	60.80 (29.67-124.61)	
Race												
White	5022 (49.6)	30.7	1 [Reference]	1 [Reference]	5.2	1 [Reference]	1 [Reference]		12.2	1 [Reference]	1 [Reference]	
Black	4215 (41.6)	39.9	1.30 (1.20-1.40)	1.13 (1.04-1.23)	8.9	1.64 (1.37-1.97)	1.52 (1.25-1.85)		9.6	1.09 (0.93-1.26)	1.04 (0.88-1.21)	
Asian	80 (0.8)	28.8	1.17 (0.78-1.77)	1.20 (0.79-1.81)	6.3	1.68 (0.68-4.17)	2.17 (0.87-5.45)		7.5	1.67 (0.72-3.87)	1.99 (0.85-4.65)	
American Indian or American Native, Native Hawaiian or Pacific Islander	140 (1.4)	21.5	0.74 (0.51-1.06)	0.74 (0.52-1.06)	8.0	1.81 (0.97-3.36)	1.69 (0.90-3.19)		11.5	1.59 (0.95-2.66)	1.67 (0.99-2.82)	
Missing or unknown	674 (6.7)	28.4	0.97 (0.83-1.13)	1.03 (0.87-1.22)	4.5	0.96 (0.65-1.41)	1.08 (0.71-1.66)		8.2	0.90 (0.69-1.19)	1.06 (0.78-1.44)	
Ethnicity												
Non-Hispanic	8876 (87.6)	34.9	1 [Reference]	1 [Reference]	6.9	1 [Reference]	1 [Reference]		11.3	1 [Reference]	1 [Reference]	
Hispanic	944 (9.3)	30.8	1.04 (0.91-1.19)	1.08 (0.94-1.24)	5.7	0.97 (0.72-1.33)	1.09 (0.78-1.52)		7.5	1.05 (0.81-1.36)	1.03 (0.79-1.35)	
Missing or unknown	311 (3.1)	26.1	0.80 (0.64-1.00)	0.96 (0.75-1.23)	4.5	0.81 (0.48-1.39)	1.06 (0.58-1.92)		6.5	0.63 (0.41-0.98)	0.63 (0.39-1.03)	
COVID-19 related deaths per million residents^b^												
<130	1925 (19.0)	35.6	1 [Reference]	1 [Reference]	6.8	1 [Reference]	1 [Reference]		9.8	1 [Reference]	1 [Reference]	
130-350	2359 (23.3)	32.6	0.94 (0.75-1.17)	0.90 (0.81-1.00)	5.5	0.68 (0.41-1.14)	0.81 (0.63-1.04)		9.9	0.67 (0.46-0.98)	1.13 (0.93-1.37)	
350-700	2629 (26.0)	37.4	0.83 (0.65-1.06)	0.89 (0.80-0.98)	8.2	0.99 (0.56-1.72)	0.96 (0.76-1.20)		9.5	0.67 (0.44-1.01)	1.02 (0.84-1.24)	
≥700	3218 (31.8)	32.1	0.92 (0.69-1.22)	0.79 (0.72-0.87)	6.4	1.22 (0.63-2.35)	0.90 (0.72-1.13)		13.1	0.68 (0.44-1.05)	1.21 (1.02-1.45)	
Urban vs rural												
Rural or highly rural	2412 (23.8)	26.7	1 [Reference]	1 [Reference]	5.4	1 [Reference]	1 [Reference]		10.2	1 [Reference]	1 [Reference]	
Urban	7714 (76.1)	36.6	1.28 (1.16-1.41)	1.17 (1.07-1.28)	7.1	1.25 (1.00-1.56)	1.10 (0.91-1.35)		11.0	1.01 (0.85-1.20)	0.92 (0.80-1.07)	
BMI at index date												
18.5-24.9, indicating normal weight	1889 (18.6)	43.3	1 [Reference]	1 [Reference]	6.5	1 [Reference]	1 [Reference]		16.2	1 [Reference]	1 [Reference]	
<18.5, indicating underweight	281 (2.8)	57.5	1.25 (1.05-1.48)	1.19 (1.00-1.42)	6.8	1.01 (0.63-1.62)	0.90 (0.56-1.46)		22.9	1.32 (1.01-1.74)	1.29 (0.98-1.70)	
25.0-29.9, indicating overweight	3167 (31.3)	33.2	0.83 (0.76-0.91)	0.84 (0.77-0.93)	6.5	1.14 (0.91-1.43)	1.04 (0.82-1.31)		10.6	0.91 (0.77-1.06)	0.90 (0.77-1.06)	
30.0-34.9, indicating obesity I	2574 (25.4)	30.2	0.81 (0.74-0.90)	0.80 (0.72-0.89)	6.4	1.23 (0.97-1.57)	1.03 (0.80-1.33)		7.8	0.86 (0.71-1.03)	0.84 (0.69-1.01)	
≥35, indicating obesity II or III	1968 (19.4)	32.2	0.94 (0.84-1.05)	0.87 (0.77-0.98)	8.0	1.71 (1.33-2.20)	1.22 (0.93-1.61)		7.9	1.12 (0.91-1.37)	0.97 (0.77-1.21)	
Missing	252 (2.5)	11.9	0.37 (0.26-0.53)	0.49 (0.34-0.71)	3.3	0.74 (0.36-1.52)	1.08 (0.52-2.27)		12.1	0.81 (0.55-1.20)	0.86 (0.57-1.30)	

Abbreviations: BMI, body mass index (calculated as weight in kilograms divided by height in meters squared); COVID-19, coronavirus disease 2019; NA, not applicable; SARS-CoV-2, severe acute respiratory syndrome coronavirus 2.

^a^ Adjusted for all sociodemographic characteristics, comorbid conditions, and symptoms listed in Table 1, Table 2, and Table 3 and stratified by station.

^b^ Categorized according to the number of COVID-19 related deaths per million reported by each state as of June 11, 2020,^27^ categorized as less than 130 per 1 million for Alaska, Arkansas, California, Hawaii, Idaho, Kansas, Kentucky, Maine, Montana, North Carolina, North Dakota, Nebraska, Oklahoma, Oregon, Puerto Rico, South Carolina, South Dakota, Tennessee, Texas, Utah, Vermont, Wisconsin, West Virginia, and Wyoming; 130 to 350 per 1 million for Alabama, Arizona, Colorado, Florida, Georgia, Iowa, Minnesota, Missouri, Mississippi, New Hampshire, New Mexico, Nevada, Ohio, Virginia, and Washington; 350 to 700 per 1 million for Delaware, Illinois, Indiana, Louisiana, Maryland, Michigan, and Pennsylvania; and more than 700 per 1 million for Connecticut, Massachusetts, New Jersey, New York, and Rhode Island. These analyses were not stratified by station to avoid geographical overadjustment.

Comorbid conditions were extracted by VINCI analysts based on *International Classification of Diseases and Related Health Problems, Tenth Revision *(*ICD-10*) recorded in VA electronic health records during the 2-year period on or before the index date.^25^ We used the Charlson Comorbidity Index (CCI) to estimate the overall burden of comorbidity (Table 2).

**Table 2.** Associations Between Comorbid Conditions and Hospitalization, Mechanical Ventilation, or Mortality Among 10 131 VA Patients Who Tested Positive for SARS-CoV-2 Between February 28 and May 14, 2020

Condition	Patients, No. (%)	Hospitalization			Mechanical ventilation			Mortality		
		30-d Rate, %	Hazard ratio (95% CI)		30-d Rate, %	Hazard ratio (95% CI)		30-d Rate, %	Hazard ratio (95% CI)	
			Age-adjusted	Adjusted^a^		Age-adjusted	Adjusted^a^		Age-adjusted	Adjusted^a^
Diabetes										
No	6270 (61.9)	28.4	1 [Reference]	1 [Reference]	4.5	1 [Reference]	1 [Reference]	8.9	1 [Reference]	1 [Reference]
Yes	3861 (38.1)	43.8	1.31 (1.23-1.41)	1.17 (1.08-1.26)	10.3	1.73 (1.48-2.02)	1.40 (1.18-1.67)	13.8	1.22 (1.08-1.38)	1.13 (0.99-1.29)
Cancer										
No	7835 (77.3)	31.7	1 [Reference]	1 [Reference]	6.0	1 [Reference]	1 [Reference]	10.0	1 [Reference]	1 [Reference]
Yes	2296 (22.7)	43.0	1.13 (1.05-1.22)	0.98 (0.91-1.06)	9.0	1.15 (0.97-1.36)	0.99 (0.84-1.18)	13.4	0.98 (0.86-1.12)	0.92 (0.80-1.05)
Hypertension										
No	3837 (37.9)	22.7	1 [Reference]	1 [Reference]	3.1	1 [Reference]	1 [Reference]	7.5	1 [Reference]	1 [Reference]
Yes	6294 (62.1)	41.3	1.40 (1.29-1.52)	1.15 (1.05-1.26)	8.9	1.84 (1.50-2.26)	1.30 (1.03-1.64)	12.8	1.05 (0.91-1.21)	0.95 (0.81-1.12)
Coronary artery disease										
No	7928 (78.3)	30.9	1 [Reference]	1 [Reference]	5.8	1 [Reference]	1 [Reference]	8.9	1 [Reference]	1 [Reference]
Yes	2203 (21.7)	46.4	1.23 (1.14-1.33)	1.04 (0.95-1.13)	10.0	1.27 (1.07-1.50)	0.95 (0.78-1.15)	17.5	1.18 (1.04-1.34)	1.02 (0.88-1.18)
Congestive heart failure										
No	9006 (88.9)	31.7	1 [Reference]	1 [Reference]	5.9	1 [Reference]	1 [Reference]	9.3	1 [Reference]	1 [Reference]
Yes	1125 (11.1)	55.1	1.45 (1.32-1.59)	1.05 (0.95-1.17)	13.2	1.68 (1.39-2.04)	1.08 (0.86-1.36)	22.8	1.54 (1.33-1.78)	1.30 (1.10-1.54)
Cerebrovascular disease										
No	9770 (96.4)	33.8	1 [Reference]	1 [Reference]	6.6	1 [Reference]	1 [Reference]	10.4	1 [Reference]	1 [Reference]
Yes	361 (3.6)	47.7	1.16 (1.00-1.36)	1.00 (0.86-1.18)	9.2	1.07 (0.75-1.54)	0.92 (0.63-1.32)	21.2	1.31 (1.04-1.67)	1.22 (0.96-1.55)
Dialysis										
No	9786 (96.6)	33.4	1 [Reference]	1 [Reference]	6.6	1 [Reference]	1 [Reference]	10.6	1 [Reference]	1 [Reference]
Yes	345 (3.4)	59.3	1.53 (1.32-1.76)	1.06 (0.91-1.24)	10.4	1.18 (0.84-1.65)	0.76 (0.52-1.09)	16.5	1.23 (0.94-1.62)	0.83 (0.62-1.11)
Chronic kidney disease										
No	8264 (81.6)	30.0	1 [Reference]	1 [Reference]	5.6	1 [Reference]	1 [Reference]	8.8	1 [Reference]	1 [Reference]
Yes	1867 (18.4)	53.3	1.49 (1.38-1.61)	1.21 (1.11-1.32)	11.7	1.57 (1.32-1.85)	1.16 (0.96-1.41)	19.5	1.41 (1.24-1.61)	1.25 (1.08-1.45)
Cirrhosis										
No	9836 (97.1)	33.7	1 [Reference]	1 [Reference]	6.6	1 [Reference]	1 [Reference]	10.6	1 [Reference]	1 [Reference]
Yes	295 (2.9)	53.5	1.47 (1.25-1.72)	1.27 (1.08-1.49)	12.0	1.54 (1.09-2.19)	1.39 (0.97-2.00)	16.9	1.76 (1.33-2.34)	1.55 (1.16-2.07)
Asthma										
No	9386 (92.6)	34.3	1 [Reference]	1 [Reference]	6.6	1 [Reference]	1 [Reference]	11.0	1 [Reference]	1 [Reference]
Yes	745 (7.4)	33.9	1.08 (0.95-1.22)	0.99 (0.87-1.13)	7.7	1.29 (0.99-1.69)	1.06 (0.80-1.41)	7.8	0.85 (0.65-1.11)	0.80 (0.60-1.05)
COPD										
No	8228 (81.2)	31.3	1 [Reference]	1 [Reference]	5.8	1 [Reference]	1 [Reference]	9.5	1 [Reference]	1 [Reference]
Yes	1903 (18.8)	47.0	1.27 (1.17-1.37)	1.05 (0.96-1.14)	10.7	1.44 (1.21-1.71)	1.20 (0.99-1.45)	16.3	1.15 (1.00-1.32)	1.02 (0.88-1.19)
Obstructive sleep apnea										
No	7411 (73.2)	32.9	1 [Reference]	1 [Reference]	5.8	1 [Reference]	1 [Reference]	11.2	1 [Reference]	1 [Reference]
Yes	2720 (26.8)	38.0	1.21 (1.13-1.31)	1.07 (0.99-1.17)	9.2	1.64 (1.39-1.93)	1.22 (1.01-1.46)	9.6	1.19 (1.03-1.37)	1.11 (0.94-1.30)
Obesity hypoventilation										
No	10 053 (99.2)	34.1	1 [Reference]	1 [Reference]	6.6	1 [Reference]	1 [Reference]	10.7	1 [Reference]	1 [Reference]
Yes	78 (0.8)	52.8	1.46 (1.07-1.99)	1.20 (0.87-1.65)	26.2	3.15 (1.98-5.00)	1.99 (1.19-3.31)	23.4	2.23 (1.38-3.62)	1.66 (0.99-2.77)
Alcohol dependence										
No	9041 (89.2)	33.5	1 [Reference]	1 [Reference]	6.8	1 [Reference]	1 [Reference]	11.2	1 [Reference]	1 [Reference]
Yes	1090 (10.8)	40.3	1.36 (1.23-1.51)	1.24 (1.11-1.39)	5.8	0.95 (0.73-1.23)	1.05 (0.79-1.39)	7.6	1.01 (0.80-1.26)	1.04 (0.82-1.32)
Hyperlipidemia										
No	4501 (44.4)	28.3	1 [Reference]	1 [Reference]	4.8	1 [Reference]	1 [Reference]	9.2	1 [Reference]	1 [Reference]
Yes	5630 (55.6)	39.0	1.14 (1.06-1.23)	0.98 (0.90-1.06)	8.2	1.26 (1.06-1.49)	0.94 (0.78-1.13)	12.1	1.02 (0.90-1.16)	0.96 (0.83-1.11)
Smoking										
Never	3644 (36.0)	29.9	1 [Reference]	1 [Reference]	6.0	1 [Reference]	1 [Reference]	8.3	1 [Reference]	1 [Reference]
Former	4077 (40.2)	38.7	1.08 (1.00-1.17)	1.01 (0.94-1.10)	8.5	1.11 (0.93-1.32)	1.02 (0.85-1.22)	12.8	1.08 (0.93-1.25)	1.02 (0.88-1.19)
Current	1135 (11.2)	36.1	1.17 (1.04-1.31)	1.10 (0.98-1.25)	5.3	0.81 (0.60-1.07)	0.94 (0.69-1.28)	7.2	0.87 (0.68-1.11)	0.87 (0.67-1.13)
Unknown	1275 (12.6)	30.7	0.95 (0.84-1.07)	1.21 (1.06-1.38)	4.5	0.73 (0.54-0.99)	1.04 (0.75-1.43)	14.5	1.22 (1.01-1.48)	1.32 (1.07-1.63)
Charlson Comorbidity Index score^b^										
0	3139 (31.0)	18.8	1 [Reference]	1 [Reference]	2.7	1 [Reference]	1 [Reference]	4.5	1 [Reference]	1 [Reference]
1-2	3023 (29.8)	31.7	1.39 (1.25-1.55)	1.32 (1.18-1.47)	6.2	1.75 (1.34-2.28)	1.54 (1.17-2.04)	9.4	1.30 (1.06-1.60)	1.40 (1.12-1.74)
3-4	1784 (17.6)	42.3	1.76 (1.57-1.97)	1.61 (1.42-1.82)	8.4	2.15 (1.62-2.85)	1.86 (1.38-2.51)	14.3	1.52 (1.23-1.89)	1.64 (1.30-2.07)
≥5	2185 (21.6)	53.6	2.17 (1.94-2.42)	1.82 (1.61-2.05)	12.1	2.83 (2.17-3.70)	2.15 (1.61-2.87)	18.9	1.89 (1.55-2.31)	1.93 (1.54-2.42)

Abbreviations: COPD, chronic obstructive pulmonary disease; SARS-CoV-2, severe acute respiratory syndrome coronavirus 2; VA, Veterans Health Administration.

^a^ Adjusted for all sociodemographic characteristics, comorbid conditions, and symptoms listed in Table 1, Table 2, and Table 3 and stratified by station.

^b^ Individual comorbid conditions were not adjusted for.

We also included documented symptoms thought to be related to SARS-CoV-2, identified by VINCI analysts based on a combination of natural language processing of text notes in patients’ electronic medical records and searching for relevant *ICD-10* codes,^25^ occurring on or within 30 days prior to the index date (Table 3). We do not report associations with loss of smell or taste, given that these symptoms were not widely recognized during the ascertainment period and thus rarely reported.

**Table 3.** Associations Between Symptoms and Hospitalization, Mechanical Ventilation, or Mortality Among 10 131 Patients Who Tested Positive for SARS-CoV-2 Between February 28 and May 14, 2020

Symptom	Patients, No. (%)	Hospitalization			Mechanical ventilation			Mortality		
		30-d Rate, %	Hazard ratio (95% CI)		30-d Rate, %	Hazard ratio (95% CI)		30-d Rate, %	Hazard ratio (95% CI)	
			Age-adjusted	Adjusted^a^		Age-adjusted	Adjusted^a^		Age-adjusted	Adjusted^a^
**Constitutional**										
Fever										
No	5944 (58.7)	24.1	1 [Reference]	1 [Reference]	3.8	1 [Reference]	1 [Reference]	9.8	1 [Reference]	1 [Reference]
Yes	4187 (41.3)	48.6	2.22 (2.07-2.38)	1.91 (1.78-2.06)	10.8	2.83 (2.40-3.33)	2.31 (1.95-2.75)	12.2	1.54 (1.36-1.74)	1.51 (1.32-1.72)
Cold										
No	8735 (86.2)	34.2	1 [Reference]	1 [Reference]	6.6	1 [Reference]	1 [Reference]	11.5	1 [Reference]	1 [Reference]
Yes	1396 (13.8)	34.2	1.02 (0.93-1.13)	0.86 (0.77-0.95)	7.2	1.10 (0.88-1.36)	0.86 (0.69-1.08)	6.0	0.72 (0.57-0.91)	0.69 (0.54-0.87)
Chills										
No	9838 (97.1)	34.0	1 [Reference]	1 [Reference]	6.7	1 [Reference]	1 [Reference]	10.8	1 [Reference]	1 [Reference]
Yes	293 (2.9)	44.0	1.32 (1.11-1.58)	1.01 (0.85-1.21)	8.2	1.20 (0.80-1.81)	0.84 (0.55-1.28)	8.9	1.08 (0.73-1.60)	1.07 (0.72-1.60)
Myalgia										
No	9934 (98.1)	34.4	1 [Reference]	1 [Reference]	6.8	1 [Reference]	1 [Reference]	11.0	1 [Reference]	1 [Reference]
Yes	197 (1.9)	26.4	0.89 (0.68-1.17)	0.70 (0.53-0.93)	2.6	0.40 (0.17-0.97)	0.32 (0.13-0.79)	2.0	0.35 (0.13-0.93)	0.37 (0.14-1.00)
Fatigue										
No	9229 (91.1)	31.7	1 [Reference]	1 [Reference]	6.3	1 [Reference]	1 [Reference]	10.3	1 [Reference]	1 [Reference]
Yes	902 (8.9)	60.8	1.69 (1.54-1.86)	1.32 (1.20-1.46)	11.2	1.41 (1.14-1.75)	1.07 (0.85-1.33)	16.1	1.15 (0.96-1.38)	1.03 (0.86-1.24)
**Respiratory**										
Cough										
No	7511 (74.1)	32.6	1 [Reference]	1 [Reference]	6.2	1 [Reference]	1 [Reference]	12.0	1 [Reference]	1 [Reference]
Yes	2620 (25.9)	38.8	1.29 (1.19-1.39)	0.90 (0.83-0.97)	8.1	1.30 (1.10-1.54)	0.78 (0.65-0.93)	7.4	0.84 (0.72-0.99)	0.69 (0.58-0.82)
Dyspnea										
No	8224 (81.2)	27.3	1 [Reference]	1 [Reference]	4.4	1 [Reference]	1 [Reference]	9.8	1 [Reference]	1 [Reference]
Yes	1907 (18.8)	64.0	2.49 (2.31-2.67)	2.18 (2.02-2.36)	16.9	3.56 (3.04-4.16)	2.95 (2.49-3.49)	15.2	1.76 (1.53-2.03)	1.78 (1.53-2.07)
Sore throat										
No	10 017 (98.9)	34.3	1 [Reference]	1 [Reference]	6.7	1 [Reference]	1 [Reference]	10.9	1 [Reference]	1 [Reference]
Yes	114 (1.1)	30.8	1.09 (0.78-1.52)	1.05 (0.75-1.46)	7.0	1.27 (0.62-2.56)	1.31 (0.63-2.72)	1.8	0.30 (0.08-1.23)	0.38 (0.09-1.56)
**Gastrointestinal**										
Nausea										
No	9801 (96.7)	33.4	1 [Reference]	1 [Reference]	6.5	1 [Reference]	1 [Reference]	10.8	1 [Reference]	1 [Reference]
Yes	330 (3.3)	60.1	1.94 (1.68-2.25)	1.43 (1.23-1.67)	12.8	1.91 (1.39-2.62)	1.56 (1.11-2.19)	11.1	1.22 (0.87-1.70)	1.21 (0.85-1.72)
Diarrhea										
No	9585 (94.6)	33.0	1 [Reference]	1 [Reference]	6.3	1 [Reference]	1 [Reference]	10.8	1 [Reference]	1 [Reference]
Yes	546 (5.4)	56.6	1.79 (1.59-2.02)	1.29 (1.14-1.46)	13.9	2.05 (1.61-2.62)	1.57 (1.21-2.02)	10.3	1.09 (0.83-1.42)	1.02 (0.77-1.35)
Abdominal pain										
No	9851 (97.2)	33.5	1 [Reference]	1 [Reference]	6.6	1 [Reference]	1 [Reference]	10.8	1 [Reference]	1 [Reference]
Yes	280 (2.8)	61.8	1.82 (1.56-2.12)	1.39 (1.19-1.63)	9.8	1.21 (0.82-1.78)	0.90 (0.60-1.35)	9.8	0.86 (0.58-1.26)	0.76 (0.51-1.13)
**Neurological**										
Headache										
No	9784 (96.6)	34.4	1 [Reference]	1 [Reference]	6.8	1 [Reference]	1 [Reference]	11.0	1 [Reference]	1 [Reference]
Yes	347 (3.4)	30.9	1.08 (0.89-1.31)	0.90 (0.74-1.10)	4.9	0.85 (0.52-1.38)	0.67 (0.41-1.09)	4.0	0.70 (0.41-1.19)	0.73 (0.42-1.24)

Abbreviation: SARS-CoV-2, severe acute respiratory syndrome coronavirus 2.

^a^ Adjusted for all sociodemographic characteristics, comorbid conditions, and symptoms listed in Table 1, Table 2, and Table 3 and stratified by station.

We analyzed 13 routinely available laboratory blood tests (Table 4). For each test, we extracted the value closest to the index date, on or within 10 days before the index date, or, if absent, within 5 days after the index date (2539 of 2905 [87.4%] were performed within 2 days of the index date).

**Table 4.** Associations Between Selected Laboratory Test Results and Mechanical Ventilation or Mortality Among 2905 VA Patients Who Tested Positive for SARS-CoV-2 and Were Hospitalized Between February 28 and May 14, 2020

Test result	Patients, No. (%)	Mechanical ventilation			Mortality		
		30-d Rate, %	Hazard ratio (95% CI)		30-d Rate, (%)	Hazard ratio (95% CI)	
			Age-adjusted	Adjusted^a^		Age-adjusted	Adjusted^a^
All patients	2905 (100)	21.2	NA	NA	21.3	NA	NA
Albumin, g/dL							
>3.9	607 (20.9)	18.7	1 [Reference]	1 [Reference]	16.3	1 [Reference]	1 [Reference]
>3.5 to 3.9	671 (23.1)	20.5	1.00 (0.77-1.30)	0.94 (0.72-1.23)	19.0	1.07 (0.82-1.41)	1.00 (0.75-1.32)
>3.1 to 3.5	673 (23.2)	23.8	1.27 (0.98-1.66)	1.23 (0.93-1.61)	22.3	1.17 (0.89-1.54)	1.09 (0.82-1.44)
>2.7 to 3.1	455 (15.7)	21.4	1.19 (0.88-1.62)	1.17 (0.86-1.61)	26.1	1.45 (1.07-1.96)	1.33 (0.97-1.81)
≤2.7	315 (10.8)	29.3	1.78 (1.29-2.45)	1.90 (1.36-2.67)	30.6	2.19 (1.58-3.03)	2.05 (1.46-2.88)
Missing	184 (6.3)	7.3	0.30 (0.15-0.60)	0.34 (0.17-0.69)	12.6	0.67 (0.38-1.19)	0.66 (0.37-1.18)
ALT, U/L							
≤18	696 (24.0)	15.6	1 [Reference]	1 [Reference]	20.6	1 [Reference]	1 [Reference]
>18 to 28	700 (24.1)	20.6	1.28 (0.99-1.65)	1.23 (0.95-1.60)	23.1	1.35 (1.07-1.70)	1.38 (1.09-1.76)
>28 to 44	652 (22.4)	25.4	1.75 (1.36-2.25)	1.65 (1.27-2.15)	21.0	1.30 (1.02-1.66)	1.39 (1.08-1.80)
>44 to 68	387 (13.3)	28.3	2.07 (1.57-2.74)	1.86 (1.39-2.49)	22.3	1.67 (1.27-2.20)	1.74 (1.30-2.32)
>68	270 (9.3)	26.3	1.90 (1.39-2.60)	1.74 (1.26-2.41)	22.6	1.76 (1.29-2.41)	1.86 (1.35-2.57)
Missing	200 (6.9)	7.7	0.24 (0.11-0.56)	0.26 (0.11-0.62)	13.3	0.53 (0.28-1.00)	0.53 (0.28-1.01)
AST, U/L							
≤25	688 (23.7)	13.2	1 [Reference]	1 [Reference]	15.2	1 [Reference]	1 [Reference]
>25 to 37	687 (23.6)	15.5	1.23 (0.92-1.63)	1.21 (0.91-1.62)	18.7	1.24 (0.95-1.61)	1.29 (0.98-1.68)
>37 to 57	672 (23.1)	27.3	2.25 (1.74-2.92)	2.20 (1.69-2.88)	22.5	1.67 (1.30-2.16)	1.74 (1.34-2.26)
>57 to 89	395 (13.6)	33.5	2.92 (2.22-3.85)	2.76 (2.07-3.68)	30.0	2.28 (1.74-2.99)	2.34 (1.77-3.10)
>89	261 (9.0)	33.1	3.09 (2.28-4.20)	2.92 (2.13-4.02)	33.7	2.82 (2.10-3.78)	3.00 (2.21-4.07)
Missing	202 (7.0)	7.2	0.42 (0.22-0.81)	0.46 (0.24-0.90)	12.0	0.78 (0.46-1.33)	0.80 (0.47-1.38)
Creatinine, mg/dL							
≤0.98	697 (24.0)	13.2	1 [Reference]	1 [Reference]	13.6	1 [Reference]	1 [Reference]
>0.98 to 1.24	686 (23.6)	18.4	1.43 (1.08-1.88)	1.35 (1.01-1.79)	14.9	1.19 (0.89-1.58)	1.22 (0.91-1.64)
>1.24 to 1.82	689 (23.7)	23.0	1.79 (1.37-2.34)	1.75 (1.32-2.32)	23.9	1.72 (1.32-2.24)	1.87 (1.42-2.47)
>1.82 to 3.80	414 (14.3)	36.8	2.90 (2.21-3.81)	3.24 (2.38-4.41)	36.3	2.62 (2.00-3.43)	3.05 (2.26-4.11)
>3.80	275 (9.5)	27.3	2.07 (1.51-2.84)	3.30 (2.25-4.84)	30.2	2.31 (1.70-3.14)	3.79 (2.62-5.48)
Missing	144 (5.0)	8.5	0.27 (0.09-0.85)	0.39 (0.13-1.23)	14.3	1.33 (0.59-3.01)	1.57 (0.70-3.52)
White blood cell count, /μL							
≤4770	712 (24.5)	16.3	1 [Reference]	1 [Reference]	17.2	1 [Reference]	1 [Reference]
>4770 to 6200	716 (24.6)	21.1	1.35 (1.05-1.73)	1.36 (1.06-1.76)	19.9	1.15 (0.90-1.48)	1.17 (0.91-1.50)
>6200 to 8300	707 (24.3)	21.2	1.37 (1.07-1.75)	1.41 (1.09-1.81)	22.0	1.20 (0.94-1.53)	1.23 (0.96-1.57)
>8300 to 11 220	424 (14.6)	24.6	1.61 (1.22-2.11)	1.74 (1.32-2.30)	22.6	1.25 (0.95-1.64)	1.28 (0.97-1.69)
>11 220	284 (9.8)	30.2	2.02 (1.52-2.70)	2.34 (1.74-3.14)	32.2	2.04 (1.54-2.70)	2.16 (1.62-2.87)
Missing	62 (2.1)	11.7	NA	NA	16.4	2.17 (0.84-5.56)	2.62 (0.99-6.91)
Neutrophil count, /μL							
≤3180	695 (23.9)	16.6	1 [Reference]	1 [Reference]	17.6	1 [Reference]	1 [Reference]
>3180 to 4500	702 (24.2)	18.2	1.06 (0.82-1.37)	1.04 (0.80-1.35)	17.9	0.99 (0.76-1.28)	1.00 (0.77-1.30)
>4500 to 6610	687 (23.6)	24.3	1.53 (1.20-1.96)	1.51 (1.18-1.93)	23.6	1.25 (0.99-1.60)	1.27 (0.99-1.62)
>6610 to 10 140	417 (14.4)	24.1	1.42 (1.08-1.87)	1.50 (1.13-1.98)	23.9	1.27 (0.97-1.66)	1.29 (0.98-1.70)
>10 140	277 (9.5)	29.1	2.25 (1.64-3.11)	2.65 (1.90-3.69)	29.7	2.01 (1.47-2.74)	2.03 (1.47-2.80)
Missing	127 (4.4)	18.5	1.05 (0.61-1.80)	1.18 (0.68-2.04)	20.6	1.69 (1.05-2.74)	1.75 (1.07-2.86)
Lymphocyte count, /μL							
>1400	663 (22.8)	13.3	1 [Reference]	1 [Reference]	12.2	1 [Reference]	1 [Reference]
>990 to 1400	693 (23.9)	21.8	1.77 (1.33-2.36)	1.67 (1.25-2.24)	19.2	1.48 (1.10-1.99)	1.44 (1.07-1.95)
>700 to 990	596 (20.5)	22.9	1.87 (1.39-2.51)	1.74 (1.29-2.35)	22.6	1.67 (1.24-2.25)	1.72 (1.27-2.33)
>500 to 700	404 (13.9)	25.4	2.01 (1.47-2.74)	1.95 (1.42-2.67)	27.8	2.14 (1.57-2.91)	2.14 (1.56-2.93)
≤500	374 (12.9)	27.7	2.18 (1.59-2.98)	1.98 (1.44-2.73)	30.9	2.17 (1.59-2.94)	2.00 (1.46-2.74)
Missing	175 (6.0)	19.4	1.33 (0.78-2.26)	1.41 (0.82-2.43)	23.6	2.69 (1.67-4.32)	2.69 (1.66-4.35)
Neutrophil to lymphocyte ratio							
≤2.71	700 (24.1)	12.6	1 [Reference]	1 [Reference]	12.5	1 [Reference]	1 [Reference]
>2.71 to 4.56	699 (24.1)	19.0	1.59 (1.20-2.11)	1.50 (1.13-2.00)	18.9	1.53 (1.16-2.03)	1.48 (1.11-1.96)
>4.56 to 7.71	695 (23.9)	23.2	1.83 (1.39-2.40)	1.71 (1.30-2.27)	23.4	1.71 (1.30-2.24)	1.71 (1.29-2.25)
>7.71 to 12.70	422 (14.5)	31.4	2.72 (2.04-3.62)	2.69 (2.01-3.61)	26.7	1.88 (1.40-2.52)	1.83 (1.36-2.46)
>12.70	280 (9.6)	31.6	2.88 (2.10-3.94)	2.84 (2.06-3.92)	38.6	3.00 (2.23-4.05)	2.88 (2.12-3.91)
Missing	109 (3.8)	12.2	0.45 (0.18-1.14)	0.45 (0.18-1.14)	13.9	1.22 (0.61-2.43)	1.20 (0.59-2.45)

Abbreviations: ALT, alanine aminotransferase; AST, aspartate aminotransferase; NA, not applicable; SARS-CoV-2, severe acute respiratory syndrome coronavirus 2; VA, Veterans Health Administration.

SI conversion factors: To convert albumin to grams per liter, multiply by 10.0; ALT and AST to microkatals per liter, multiply by 0.0167; creatinine to millimoles per liter, multiply by 88.4; and lymphocyte count, neutrophil count, and white blood cell count to ×10^9^ per liter, multiply by 0.001.

^a^ Adjusted for all sociodemographic characteristics, comorbid conditions, and symptoms listed in Table 1, Table 2, and Table 3 and stratified by station.

### Statistical Analysis

Using the Kaplan-Meier method, we calculated 30-day hospitalization, mechanical ventilation, and mortality rates from the index date through June 22, 2020. Participants who did not experience the outcome of interest were censored at the end of follow-up.

All analyses were stratified by the VA medical center where patients were tested for SARS-CoV-2. We used Cox proportional hazards models to compare patients with and without SARS-CoV-2 with respect to risk of adverse outcomes. We also used Cox proportional hazards models to identify independent risk factors for each outcome among patients with SARS-CoV-2, adjusting for sociodemographic characteristics, comorbid conditions, and presenting symptoms, as listed in Table 1, Table 2, and Table 3. Laboratory tests were not included in multivariable adjustment due to concerns about overadjustment, given that these were felt to be part of the causal pathway of the disease rather than predisposing risk factors. Laboratory tests were categorized based on quintiles (ie, ≤25th, >25th to 50th, >50th to 75th, >75th to 90th, and >90th percentiles), with an additional category for missing tests, which were relatively rare. In secondary analyses, we used competing risks analysis for the outcomes of hospitalization or ventilation to account for the competing risk of death.

Multivariable population attributable fractions (PAFs) for each major risk factor were estimated by finding the mean over randomly selected permutations of the PAF when other risk factors were sequentially removed from the model. The number of permutations was sufficient to approximate the true mean to within 0.1%. Confidence intervals were calculated using Monte Carlo simulation (500 iterations over 5000 samples).

Analysis was conducted in Stata MP version 15 (StataCorp), R 64-bit version 3.6.1 (R Project for Statistical Computing), with the averisk package version 1.0.3. Statistical significance was set at *P* < .05, and all tests were 2-tailed.

## Results

Of 88 747 VA enrollees tested for SARS-CoV-2, 10 131 (11.4%) tested positive (Figure, A). Compared with individuals who tested negative, those testing positive were older (mean [SD] age, 61.6 [15.9] years vs 63.6 [16.2] years), more likely to be Black individuals (19 340 [24.6%] vs 4215 [41.6%]), more likely to have obesity (31 604 [40.2%] vs 4542 [44.8%]), and more likely to live in states with high COVID-19 burden (≥700 deaths/1 million residents: 8019 [10.2%] vs 3218 [31.8%]) but had a similar distribution of comorbid conditions and CCI scores (eTable 1 in the Supplement).

**Figure.**
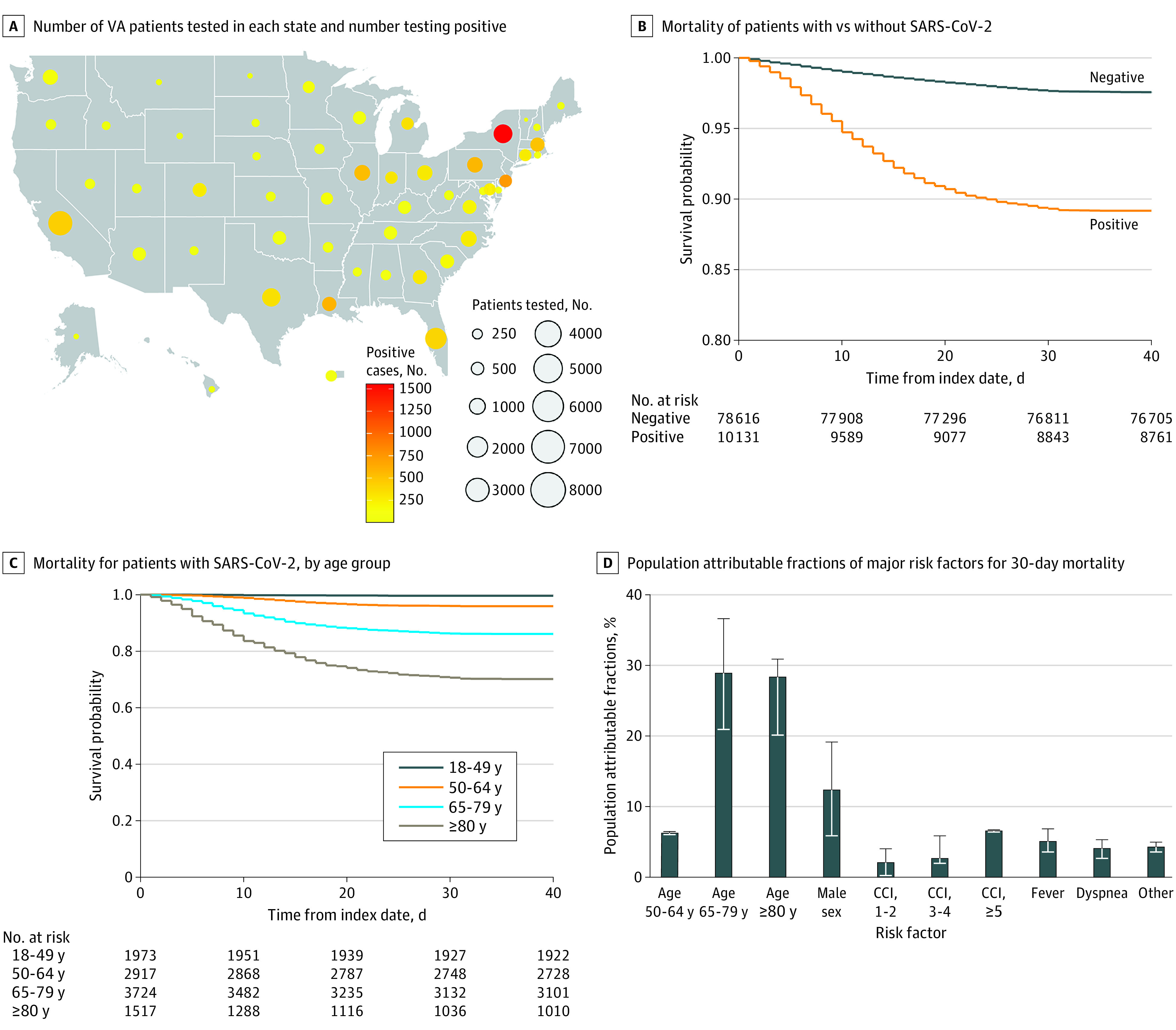
Distribution of Veterans Affairs (VA) Patients Tested for Severe Acute Respiratory Coronavirus 2 (SARS-CoV-2), Associations With Mortality, and Population Attributable Fractions (PAFs) for Major Risk Factors of Mortality D, Whiskers indicate 95% CIs. CCI indicates Charlson Comorbidity Index.

Compared with individuals who tested negative for SARS-CoV-2, those who tested positive had higher 30-day rates of hospitalization (30.4% vs 29.3%; adjusted hazard ratio [aHR], 1.13; 95% CI, 1.08-1.13), mechanical ventilation (6.7% vs 1.7%; aHR, 4.15; 95% CI, 3.74-4.61), and mortality (10.8% vs 2.4%; aHR, 4.44; 95% CI, 4.07-4.83) after adjusting for sociodemographic characteristics and comorbid conditions (Figure, B; eTable 2 in the Supplement). Competing risks analysis (death treated as a competing risk) did not appreciably change the associations for hospitalization or ventilation (eTable 2 in the Supplement).

### Sociodemographic Characteristics and Adverse Outcomes in Patients Who Tested Positive for SARS-CoV-2

Veterans who tested positive for SARS-CoV-2 had a mean (SD) age of 63.6 (16.2) years; 9221 (91.0%) were men, 944 (9.3%) were Hispanic individuals, 5022 (49.6%) were White individuals, and 4215 (41.6%) were Black individuals (Table 1). They were more commonly from urban rather than rural areas (7714 [76.1%] vs 2412 [23.8%]) and had a high prevalence of obesity (4542 [44.8%]). They originated from all 50 US states and Puerto Rico, with the greatest number from New York (1555 [15.3%]), New Jersey (757 [7.5%]), Louisiana (598 [5.9%]), and Pennsylvania (563 [5.6%]) (Figure, A; eTable 3 in the Supplement).

Increasing age was the characteristic most strongly associated with risk of hospitalization, mechanical ventilation, and death. Compared with patients younger than 50 years of age (30-day mortality, 0.4%), those aged 50 to 64 years (30-day mortality, 4.1%; aHR, 9.27; 95% CI, 4.51-19.08), 65 to 79 years (30-day mortality, 13.8%; aHR, 27.47; 95% CI, 13.48-55.99), and 80 years and older (30-day mortality, 29.7%; aHR, 60.80; 95% CI, 29.67-124.61) had progressively higher mortality (Figure, C). Compared with White patients, Black patients were more likely to be hospitalized (aHR, 1.13; 95% CI, 1.04-1.23) and to receive mechanical ventilation (aHR, 1.52; 95% CI, 1.25-1.85) but no more likely to die (aHR, 1.04; 95% CI, 0.88-1.21). Compared with women, men were likely to be hospitalized (aHR, 1.22; 95% CI, 1.04-1.42) or to receive mechanical ventilation (aHR, 2.07; 95% CI, 1.30-3.32), but the association of male sex with mortality did not reach statistical significance (aHR, 1.38; 95% CI, 0.93-2.06), likely reflecting the small number of women in the sample. Areas with high regional COVID-19 disease burden were associated with increased risk of death (eg, ≥700 vs <130 deaths per 1 million residents: aHR, 1.21; 95% CI, 1.02-1.45). Hispanic ethnicity (mortality: aHR, 1.03; 95% CI, 0.79-1.35), having overweight (mortality, body mass index 30.0-34.9 vs 18.5-24.9: aHR, 0.90; 95% CI, 0.77-1.06) or obesity (mortality, body mass index ≥35 vs 18.5-24.9: aHR, 0.97; 95% CI, 0.77-1.21), and urban residence (mortality: aHR, 0.92; 95% CI, 0.80-1.07) were also not associated with increased risk of adverse outcomes.

### Comorbid Conditions and Adverse Outcomes in Patients Who Tested Positive for SARS-CoV-2

Veterans who tested positive for SARS-CoV-2 had a high overall burden of comorbidity (Table 2), with less than one-third having no coexisting comorbid conditions (3139 [31.0%]). A higher CCI score was strongly associated with increasing risk of hospitalization (eg, ≥5 vs 0: aHR, 1.82; 95% CI, 1.61-2.05), mechanical ventilation (eg, ≥5 vs 0: aHR, 2.15; 95% CI, 1.61-2.87), and death (eg, ≥5 vs 0: aHR, 1.93; 95% CI, 1.54-2.42). Comorbid conditions that were significantly associated with hospitalization included diabetes (aHR, 1.17; 95% CI, 1.08-1.26), hypertension (aHR, 1.15; 95% CI, 1.05-1.26), chronic kidney disease (aHR, 1.21; 95% CI, 1.11-1.32), cirrhosis (aHR, 1.27; 95% CI, 1.08-1.49), and alcohol dependence (aHR, 1.24; 95% CI, 1.11-1.39). Comorbid conditions that were significantly associated with mechanical ventilation included diabetes (aHR, 1.40; 95% CI, 1.18-1.67), hypertension (aHR, 1.30; 95% CI, 1.03-1.64), obstructive sleep apnea (aHR, 1.22; 95% CI, 1.01-1.46), and obesity hypoventilation (aHR, 1.99; 95% CI, 1.19-3.31). Congestive heart failure (aHR, 1.30; 95% CI, 1.10-1.54), chronic kidney disease (aHR, 1.25; 95% CI, 1.08-1.45), and cirrhosis (aHR, 1.55; 95% CI, 1.16-2.07) were the only comorbid conditions significantly associated with mortality. Chronic obstructive pulmonary disease (aHR, 1.02; 95% CI, 0.88-1.19), hypertension (aHR, 0.95; 95% CI, 0.81-1.12), and smoking (eg, current vs never: aHR, 0.87; 95% CI, 0.67-1.13) were not associated with mortality.

### Documented Symptoms and Adverse Outcomes in Patients Who Tested Positive for SARS-CoV-2

The most common documented symptoms included fever (4187 [41.3%]), cough (2620 [25.9%]), and dyspnea (1907 [18.8%]) (Table 3). Symptoms that were significantly associated with hospitalization included fever (aHR, 1.91; 95% CI, 1.78-2.06), dyspnea (aHR, 2.18; 95% CI, 2.02-2.36), nausea (aHR, 1.43; 95% CI, 1.23-1.67), diarrhea (aHR, 1.29; 95% CI, 1.14-1.46), abdominal pain (aHR, 1.39; 95% CI, 1.19-1.63), and fatigue (aHR, 1.32; 95% CI, 1.20-1.46). Symptoms that were significantly associated with mechanical ventilation included fever (aHR, 2.31; 95% CI, 1.95-2.75), dyspnea (aHR, 2.95; 95% CI, 2.49-3.49), nausea (aHR 1.56; 95% CI, 1.11-2.19), and diarrhea (aHR, 1.57; 95% CI, 1.21-2.02). Only fever (aHR, 1.51; 95% CI, 1.32-1.72) and dyspnea (aHR, 1.78; 95% CI, 1.53-2.07) were significantly associated with mortality.

### Laboratory Test Results and Adverse Outcomes Among Patients Who Tested Positive for SARS-CoV-2 

Associations of laboratory tests with outcomes were only determined among 2905 hospitalized patients because they are not routinely ascertained in nonhospitalized patients. Many laboratory test results were associated with mechanical ventilation and mortality in a dose-response manner, including elevated creatinine (>3.80 mg/dL vs ≤0.98 mg/dL [to convert to millimoles per liter, multiply by 88.4], mechanical ventilation: aHR, 3.30; 95% CI, 2.25-4.84; mortality: aHR, 3.79; 95% CI, 2.62-5.48), elevated serum aspartate aminotransferase (>89 U/L vs ≤25 U/L [to convert to microkatals per liter, multiply by 0.0167], mechanical ventilation: aHR, 2.92; 95% CI, 2.13-4.02; mortality: aHR, 3.00; 95% CI, 2.21-4.07), elevated neutrophil to lymphocyte ratio (>12.70 vs ≤2.71, mechanical ventilation: aHR, 2.84; 95% CI, 2.06-3.92; mortality: aHR, 2.88; 95% CI, 2.12-3.91), elevated total white blood cell count (>11 200/μL vs ≤4770/μL [to convert to ×10^9^, multiply by 0.001], mechanical ventilation: aHR, 2.34; 95% CI, 1.74-3.14; mortality: aHR, 2.16; 95% CI, 1.62-2.87), elevated neutrophil count (>10 140/μL vs ≤3180/μL [to convert to ×10^9^, multiply by 0.001], mechanical ventilation: aHR, 2.65; 95% CI, 1.90-3.69; mortality, aHR, 2.03; 95% CI, 1.47-2.80), reduced lymphocyte count (≤500/μL vs >1400/μL [to convert to ×10^9^, multiply by 0.001], mechanical ventilation: aHR, 1.98; 95% CI, 1.44-2.73; mortality: aHR, 2.00; 95% CI, 1.46-2.74), reduced albumin (>3.9 g/dL vs ≤2.7 g/dL [to convert to grams per liter, multiply by 10.0], mechanical ventilation: aHR, 1.90; 95% CI, 1.36-2.67; mortality: aHR, 2.05; 95% CI, 1.46-2.88), and elevated alanine aminotransferase (≤18 U/L vs >68 U/L [to convert to microkatals per liter, multiply by 0.0167], mechanical ventilation: aHR, 1.74; 95% CI, 1.26-2.41; mortality, aHR, 1.86; 95% CI, 1.35-2.57) (Table 4), but not serum bilirubin, platelet count, hemoglobin, and international normalized ratio (eTable 4 in the Supplement).

### PAFs of Major Risk Factors for 30-Day Mortality

Most deaths (63.4%) were associated with older age groups relative to the reference group (ie, aged 18-49 years): 6.2% (95% CI, 6.1%-6.3%) were associated with age 50 to 64 years, 28.9% (95% CI, 20.9%-36.6%) with age 65 to 79 years, and 28.3% (95% CI, 20.1%-30.8%) with age of 80 years or older (Figure, D). Male sex (relative to female sex) contributed 12.3% (95% CI, 5.8%-19.1%). Comorbidity burden contributed 2.0% (95% CI, 0.1%-4.0%) for CCI score of 1 or 2, 2.6% (95% CI, 1.9%-5.8%) for CCI score of 3 or 4, and 6.5% (95% CI, 6.3%-6.6%) for CCI score of 5 or greater. Finally, fever contributed 5.0% (95% CI, 3.5%-6.8%) and dyspnea, 4.0% (95% CI, 2.6%-5.2%), with negligible contributions from other risk factors.

## Discussion

In a national study of 88 747 US veterans tested for SARS-CoV-2 infection between February 28 and May 14, 2020, those testing positive had a 4.2-fold risk of mechanical ventilation and a 4.4-fold risk of death compared with those testing negative. Among those who tested positive for SARS-CoV-2, older age was the strongest risk factor associated with hospitalization, mechanical ventilation, and mortality. Most deaths in this cohort were attributed to age of 50 years or older (63.4%), male sex (12.3%), and comorbidity burden, with CCI score of at least 1 (11.1%). Other risk factors for mortality included select preexisting comorbid conditions (ie, heart failure, chronic kidney disease, and cirrhosis) and presenting symptoms (ie, fever and dyspnea). Abnormal results in a range of routine laboratory tests were associated with mechanical ventilation or mortality in a dose-response manner.

Early estimates from the US Centers for Disease Control and Prevention suggested that 20.7% to 31.4% of US adults infected with SARS-CoV-2 were hospitalized.^2^ Within health systems, the percentage of patients who have been hospitalized ranged from 8% to 80.7%, depending on the clinical context of testing.^21,28,29,30,31,32^ The percentages of patients who require mechanical ventilation has ranged from 2.3% of the Chinese population to 93.2% of critically ill patients infected with SARS-CoV-2 admitted to New York area hospitals.^1,4,7,9,10,14,24,32,33,34,35,36,37,38,39,40,41,42,43,44,45,46^ Short-term mortality rates in the US population are estimated to be between 1.8% and 3.4%,^2^ which is higher than the 1.4% estimate from China earlier in the pandemic.^33^ However, short-term mortality rates in case series of hospitalized patients and high-risk populations have been much higher, ranging from 10.2% to 67%.^8,9,10,18,24,28,36,38,43,45,46,47,48,49,50,51^ Our findings demonstrating 30-day rates of hospitalization, mechanical ventilation, and death of 30.4%, 6.7%, and 10.8%, respectively, spotlight the substantial consequences of SARS-CoV-2 on the Veteran population, associated with the high prevalence of advanced age, male sex, and comorbid conditions.

Recognizing risk factors for adverse outcomes is a preliminary step toward developing prognostic models that will allow for real-time identification of patients most and least likely to benefit from available interventions (eg, close monitoring at home vs hospitalization, intensive care unit admission and mechanical ventilation, or selected therapeutics). Some risk factors may be reversible or modifiable, such that eliminating them might be a strategy for reducing the mortality rate of SARS-CoV-2 or may provide clues as to the pathogenesis of severe, life-threatening SARS-CoV-2. Risk factors that have been identified in prior studies include older age, male sex, hypertension, diabetes, chronic obstructive pulmonary disease, cardiac disease, liver disease, chronic kidney disease, neurologic disorders, cancer, obesity, higher overall burden of comorbidity, and smoking.^1,3,4,5,6,7,8,9,10,11,12,13,14,15,16,17,18,19,20,21,22,23,24^

In our cohort, older age was by far the strongest risk factor associated with ventilation and death, even after adjusting for comorbid conditions; 63.5% of deaths were attributed to being aged 50 years or older based in PAF calculations. While we observed linear associations between age and mortality, the association of age with mechanical ventilation appeared to be nonlinear, with the highest risk noted for those aged 65 to 79 years, perhaps reflecting treatment preferences and/or clinical practice. We observed strong linear associations with CCI score and all measured outcomes, suggesting that a measure of overall disease burden may be more helpful than the presence of individual comorbid conditions. PAF calculations suggested that 11.1% of deaths were attributed to having a CCI score of at least 1. Although male sex was not statistically significantly associated with mortality, 12.3% of deaths were attributed to male sex based on PAF calculations, which were statistically significant. Among hospitalized patients, abnormalities in a range of routine laboratory tests were strongly and linearly associated with ventilation and death, suggesting that these could be useful in risk stratification at the time of hospitalization.

Some risk factors for mortality reported by earlier studies did not reach statistical significance in our analyses including Black race, Hispanic ethnicity, body mass index, underlying lung disease, smoking, diabetes, and hypertension. This may reflect differences in the study population (eg, male sex, older age), differences in the confounders that were adjusted for, or attenuation of racial/ethnic disparities in access to care in the VA system relative to the private sector.^52^ Additionally, we investigated risk factors for adverse outcomes among patients who tested positive for SARS-CoV-2 rather than in the general population^53^ because the latter approach provides a composite of the risk of infection and subsequent risk of death. For example, Black and Hispanic patients may be much more likely to acquire SARS-CoV-2 but not more likely to die if infected. The finding that Black patients did not have higher mortality rates but had higher mechanical ventilation rates may be related to lower rates of advance directives in Black patients.^54^

Surprisingly, neither chronic obstructive pulmonary disease nor smoking—which are prevalent in the veteran population—were associated with adverse outcomes. Geographic burden of SARS-CoV-2 was not as strongly associated with mortality as we had anticipated. VA patients in the most highly affected states (ie, Connecticut, Massachusetts, New Jersey, New York, and Rhode Island) only had a 1.21-fold higher mortality rate than patients from the least affected states.

### Limitations

This study has limitations. Our results in the predominantly male veteran population may not be generalizable to other populations and groups, especially women. We used *ICD-10* codes for the determination of comorbid conditions. However, most *ICD-10*–based definitions have been widely used and validated in VA studies. Novel natural language processing plus *ICD-10* codes were used for the definition of SARS-CoV-2 symptoms, although the performance characteristics of these definitions are not yet known. We captured deaths that occurred both within and outside the VA; however, hospitalizations or mechanical ventilations that occurred outside the VA and were not paid for by the VA were not captured. Our results are limited to those patients who were tested within the VA system. Therefore, our results likely reflect institutional policies and practices related to testing. Strengths of our study include its national scope, large number of patients, relatively long follow-up for a range of outcomes, and analysis of many potential risk factors.

## Conclusions

In this study, we found high rates of mechanical ventilation and death among 10 131 VA patients with SARS-CoV-2 infection. Most deaths were associated with older age, male sex, and a high overall burden of comorbidity.

## Notes

Supplement. **eTable 1. **Comparison of Baseline Characteristics of Veterans Who Tested Positive (n = 10 131) vs Negative (n = 78 616) for SARS-CoV-2 **eTable 2. **Comparing Hospitalization, Mechanical Ventilation, and Mortality Rates Between Veterans Who Did and Did Not Test Positive for SARS-CoV-2 **eTable 3. **Number of Patients Who Tested Positive for SARS-CoV-2 in the VA Health Care System as of May 14, 2020, by State **eTable 4. **Associations Between Laboratory Test Results and Mechanical Ventilation or Mortality Among 2905 VA Patients Who Tested Positive for SARS-CoV-2 and Were Hospitalized Between February 28 and May 14, 2020
