# Supplementary material for: Risk Factors for Hospitalization, Mechanical Ventilation, or Death Among 10 131 US Veterans With SARS-CoV-2 Infection
Source: JAMA Netw Open. 2020 Sep 23;3(9):e2022310. doi: 10.1001/jamanetworkopen.2020.22310 (PMC7512055; doi:10.1001/jamanetworkopen.2020.22310)
Supplement: Supplement. — eTable 1. Comparison of Baseline Characteristics of Veterans Who Tested Positive (n = 10 131) vs Negative (n = 78 616) for SARS-CoV-2 eTable 2. Comparing Hospitalization, Mechanical Ventilation, and Mortality Rates Between Veterans Who Did and Did Not Test Positive for SARS-CoV-2 eTable 3. Number of Patients Who Tested Positive for SARS-CoV-2 in the VA Health Care System as of May 14, 2020, by State eTable 4. Associations Between Laboratory Test Results and Mechanical Ventilation or Mortality Among 2905 VA Patients Who Tested Positive for SARS-CoV-2 and Were Hospitalized Between February 28 and May 14, 2020 [file jamanetwopen-e2022310-s001.pdf]

Supplementary Online Content

Ioannou GN, Locke E, Green P, et al. Risk factors for hospitalization, mechanical ventilation, or death among 10 131 US veterans With SARS-CoV-2 infection. *JAMA Netw Open*. 2020;3(9):e2022310. doi:10.1001/jamanetworkopen.2020.22310

eTable 1. Comparison of Baseline Characteristics of Veterans Who Tested Positive (n = 10 131) vs Negative (n = 78 616) for SARS-CoV-2

eTable 2. Comparing Hospitalization, Mechanical Ventilation, and Mortality Rates Between Veterans Who Did and Did Not Test Positive for SARS-CoV-2

eTable 3. Number of Patients Who Tested Positive for SARS-CoV-2 in the VA Health Care System as of May 14, 2020, by State

eTable 4. Associations Between Laboratory Test Results and Mechanical Ventilation or Mortality Among 2905 VA Patients Who Tested Positive for SARS-CoV-2 and Were Hospitalized Between February 28 and May 14, 2020

This supplementary material has been provided by the authors to give readers additional information about their work.

© 2020 Ioannou GN et al. *JAMA Network Open.*

eTable 1. Comparison of Baseline Characteristics of Veterans Who Tested Positive (n = 10 131) vs Negative (n = 78 616) for SARS-CoV-2

	COVID NEGATIVE N=78,616	COVID POSITIVE N=10,131	P-value
**Sex**			< 0.001
Female	11.8	9.0	
Male	88.2	91.0	
**Age at test**, mean	61.6±15.9	63.6±16.2	< 0.001
18-49	22.5	19.5	
50-64	30.1	28.8	
65-79	36.4	36.8	
>=80	11.0	15.0	
**Race**			< 0.001
White	65.5	49.6	
Black	24.6	41.6	
Asian	1.2	0.8	
AI/AN, Native Hawaiian/Pacific Islander	1.7	1.4	
Missing/Unknow	7.0	6.7	
**Ethnicity**			< 0.001
Non-Hispanic	89.0	87.6	
Hispanic	7.6	9.3	
Missing/Unknown	3.4	3.1	
**Geographical Region** (COVID-19 deaths per million)**			< 0.001
<130	42.4	19.0	
130-350	33.6	23.3	
350-700	13.8	26.0	
>700	10.2	31.8	
**Urban/Rural**			< 0.001
Rural/Highly rural	38.4	23.8	
Urban	61.6	76.2	
**BMI at index date**			< 0.001
<18.5 (underweight)	3.1	2.8	
18.5-24.9 (normalweight)	22.4	18.6	
25.29.9 (overweight)	31.6	31.3	
30-34.9 (Obese I)	23.1	25.4	
≥35 (Obese II and III)	17.1	19.4	
Missing	2.6	2.5	
**COMORBID CONDITIONS**			
**Diabetes**			< 0.001
No	66.6	61.9	
Yes	33.4	38.1	

© 2020 Ioannou GN et al. *JAMA Network Open.*

**Cancer**			< 0.001
No	74.1	77.3	
Yes	25.9	22.7	
**Hypertension**			< 0.001
No	40.3	37.9	
Yes	59.7	62.1	
**Coronary artery disease**			< 0.001
No	76.6	78.3	
Yes	23.4	21.7	
**Congestive heart failure**			< 0.001
No	87.0	88.9	
Yes	13.0	11.1	
**Cerebrovascular Disease**			0.70
No	96.5	96.4	
Yes	3.5	3.6	
**Dialysis**			0.52
No	96.7	96.6	
Yes	3.3	3.4	
**Chronic kidney disease**			< 0.01
No	82.7	81.6	
Yes	17.3	18.4	
**Cirrhosis**			< 0.001
No	95.9	97.1	
Yes	4.1	2.9	
**Asthma**			0.46
No	92.4	92.6	
Yes	7.6	7.4	
**COPD**			< 0.001
No	75.2	81.2	
Yes	24.8	18.8	
**Obstructive Sleep Apnea**			< 0.001
No	71.1	73.2	
Yes	28.9	26.8	
**Obesity Hypoventilation**			0.73
No	99.2	99.2	
Yes	0.8	0.8	
**Alcohol Dependence**			< 0.001
No	83.5	89.2	
Yes	16.5	10.8	
**Drug Dependence**			< 0.001
No	89.8	94.2	
Yes	10.2	5.8	
**Hyperlipidemia**			0.05
No	45.5	44.4	
Yes	54.5	55.6	
**Smoking**			< 0.001

© 2020 Ioannou GN et al. *JAMA Network Open.*

Never	28.4	36.0	
Former	37.2	40.2	
Current	21.8	11.2	
Unknown	12.6	12.6	
**Charlson Comorbidity Index**			< 0.001
0	32.0	31.0	
1-2	27.2	29.8	
3-4	16.6	17.6	
>=5	24.2	21.6	
**SYMPTOMS**			
**Fever**			< 0.001
No	74.3	58.7	
Yes	25.7	41.3	
**Cold**			< 0.01
No	87.4	86.2	
Yes	12.6	13.8	
**Chills**			< 0.001
No	98.6	97.1	
Yes	1.4	2.9	
**Myalgia**			< 0.001
No	98.7	98.1	
Yes	1.3	1.9	
**Cough**			< 0.001
No	81.1	74.1	
Yes	18.9	25.9	
**Dyspnea**			0.42
No	81.5	81.2	
Yes	18.5	18.8	
**Sore throat**			< 0.001
No	98.3	98.9	
Yes	1.7	1.1	
**Nausea**			< 0.01
No	96.1	96.7	
Yes	3.9	3.3	
**Headache**			0.19
No	96.8	96.6	
Yes	3.2	3.4	
**Diarrhea**			< 0.001
No	95.5	94.6	
Yes	4.5	5.4	
**Abdominal pain**			< 0.001
No	95.2	97.2	
Yes	4.8	2.8	
**Fatigue**			< 0.001
No	92.2	91.1	
Yes	7.8	8.9	

© 2020 Ioannou GN et al. *JAMA Network Open.*

eTable 2. Comparing Hospitalization, Mechanical Ventilation, and Mortality Rates Between Veterans Who Did and Did Not Test Positive for SARS-CoV-2

SARS-CoV-2	N (%)	Event N (%)	10-day Event Rate (%)	30-day Event Rate (%)	Hazard ratio	Adjusted Hazard Ratio*†
**HOSPITALIZATION**						
Negative	78,616(88.6)	28,044(35.7)	33.2	34.4	1	1
Positive	10,131(11.4)	3,507(34.6)	32.9	34.2	1.02 (0.98-1.05)	1.09 (1.05-1.13)
**HOSPITALIZATION (with death as competing risk†)**						
Negative	78,616(88.6)	28,044(35.7)	33.2	34.4	1	1
Positive	10,131(11.4)	3,507(34.6)	32.9	34.2	0.96 (0.94-0.995)	1.01 (0.98-1.05)
**HOSPITALIZATION (OCCURRING ON OR AFTER THE SARS-COV-2 TESTING DATE)**						
Negative	72,858(88.4)	22,286(30.6)	27.9	29.3	1	1
Positive	9,573(11.6)	2,949(30.8)	29.0	30.4	1.05 (1.01-1.10)	1.13 (1.08-1.17)
**HOSPITALIZATION (OCCURRING ON OR AFTER THE SARS-COV-2 TESTING DATE) (with death as competing risk†)**						
Negative	72,858(88.4)	22,286(30.6)	27.9	29.3	1	1
Positive	9,573(11.6)	2,949(30.8)	29.0	30.4	1.00 (0.97-1.04)	1.05 (1.01-1.09)
**MECHANICAL VENTILATION**						
Negative	78,616(88.6)	1,467(1.9)	1.4	1.7	1	1
Positive	10,131(11.4)	676(6.7)	6.0	6.7	3.85 (3.48-4.26)	4.15 (3.74-4.61)
**MECHANICAL VENTILATION (with death as competing risk†)**						
Negative	78,616(88.6)	1,467(1.9)	1.4	1.7	1	1
Positive	10,131(11.4)	676(6.7)	6.0	6.7	3.66 (3.34-4.01)	4.15 (3.74-4.61)
**MORTALITY**						
Negative	78,616(88.6)	2,075(2.6)	1.1	2.4	1	1
Positive	10,131(11.4)	1,098(10.8)	5.8	10.8	4.25 (3.91-4.61)	4.44 (4.07-4.83)

* Adjusted for all the sociodemographic characteristics shown in Table 2 and the comorbidities shown in Table 3 (not adjusted for symptoms) and stratified by station.

† Competing risks analysis with death treated as a competing risk for mechanical ventilation or hospitalization.

© 2020 Ioannou GN et al. *JAMA Network Open.*

eTable 3. Number of Patients Who Tested Positive for SARS-CoV-2 in the VA Health Care System as of May 14, 2020, by State

STATE	Number of cases N=10,131	Total Number of patients tested N=88,747	Proportion of tests that were positive (%)
New York	1555	4879	31.9
New Jersey	757	1732	43.7
Louisiana	598	2393	25.0
Pennsylvania	563	2998	18.8
Illinois	536	3126	17.1
Massachusetts	512	2365	21.6
California	430	8845	4.9
Florida	397	6708	5.9
Michigan	337	1588	21.2
Texas	333	4550	7.3
Indiana	303	1612	18.8
Georgia	298	2356	12.6
Ohio	287	2833	10.1
Connecticut	273	1535	17.8
North Carolina	265	3174	8.3
Maryland	255	1503	17.0
Colorado	251	2246	11.2
Virginia	202	2233	9.0
South Carolina	171	2225	7.7
Washington	151	2802	5.4
Missouri	150	1535	9.8
Minnesota	149	1505	9.9
Arizona	125	2010	6.2
Wisconsin	116	1891	6.1
Tennessee	105	1949	5.4
Alabama	90	996	9.0
District Of Columbia	85	461	18.4
Iowa	81	963	8.4
Kentucky	74	1890	3.9
Nevada	70	973	7.2
Mississippi	68	666	10.2
Oklahoma	64	2074	3.1
Rhode Island	63	299	21.1
Delaware	57	276	20.7
Puerto Rico	56	1250	4.5
Arkansas	47	1020	4.6
Kansas	42	836	5.0
Nebraska	42	505	8.3

© 2020 Ioannou GN et al. *JAMA Network Open.*

Utah	34	757	4.5
New Mexico	33	639	5.2
New Hampshire	33	338	9.8
Oregon	31	1017	3.0
South Dakota	26	474	5.5
West Virginia	24	794	3.0
Maine	20	356	5.6
North Dakota	13	149	8.7
Hawaii	10	227	4.4
Alaska	8	159	5.0
Idaho	8	588	1.4
Vermont	6	106	5.7
Montana	6	195	3.1
Wyoming	5	244	2.0

© 2020 Ioannou GN et al. *JAMA Network Open.*

eTable 4. Associations Between Laboratory Test Results and Mechanical Ventilation or Mortality Among 2905 VA Patients Who Tested Positive for SARS-CoV-2 and Were Hospitalized Between February 28 and May 14, 2020

		MECHANICAL VENTILATION			MORTALITY		
	N (%)	Ventilation Rate (30-day) (%)	Age-adjusted Hazard Ratio	Adjusted* Hazard Ratio	Mortality Rate (30-day) (%)	Age-adjusted Hazard Ratio	Adjusted* Hazard Ratio
**Albumin**							
>3.9	607(20.9)	18.7	1	1	16.3	1	1
>3.5 to 3.9	671(23.1)	20.5	1.00 (0.77-1.30)	0.94 (0.72-1.23)	19.0	1.07 (0.82-1.41)	1.00 (0.75-1.32)
>3.1 to 3.5	673(23.2)	23.8	1.27 (0.98-1.66)	1.23 (0.93-1.61)	22.3	1.17 (0.89-1.54)	1.09 (0.82-1.44)
>2.7 to 3.1	455(15.7)	21.4	1.19 (0.88-1.62)	1.17 (0.86-1.61)	26.1	1.45 (1.07-1.96)	1.33 (0.97-1.81)
<=2.7	315(10.8)	29.3	1.78 (1.29-2.45)	1.90 (1.36-2.67)	30.6	2.19 (1.58-3.03)	2.05 (1.46-2.88)
Missing	184(6.3)	7.3	0.30 (0.15-0.60)	0.34 (0.17-0.69)	12.6	0.67 (0.38-1.19)	0.66 (0.37-1.18)
**ALT**							
<=18	696(24.0)	15.6	1	1	20.6	1	1
>18 to 28	700(24.1)	20.6	1.28 (0.99-1.65)	1.23 (0.95-1.60)	23.1	1.35 (1.07-1.70)	1.38 (1.09-1.76)
>28 to 44	652(22.4)	25.4	1.75 (1.36-2.25)	1.65 (1.27-2.15)	21.0	1.30 (1.02-1.66)	1.39 (1.08-1.80)
>44 to 68	387(13.3)	28.3	2.07 (1.57-2.74)	1.86 (1.39-2.49)	22.3	1.67 (1.27-2.20)	1.74 (1.30-2.32)
>68	270(9.3)	26.3	1.90 (1.39-2.60)	1.74 (1.26-2.41)	22.6	1.76 (1.29-2.41)	1.86 (1.35-2.57)
Missing	200(6.9)	7.7	0.24 (0.11-0.56)	0.26 (0.11-0.62)	13.3	0.53 (0.28-1.00)	0.53 (0.28-1.01)
**AST**							
<=25	688(23.7)	13.2	1	1	15.2	1	1
>25 to 37	687(23.6)	15.5	1.23 (0.92-1.63)	1.21 (0.91-1.62)	18.7	1.24 (0.95-1.61)	1.29 (0.98-1.68)
>40 to 57	672(23.1)	27.3	2.25 (1.74-2.92)	2.20 (1.69-2.88)	22.5	1.67 (1.30-2.16)	1.74 (1.34-2.26)
>57 to 89	395(13.6)	33.5	2.92 (2.22-3.85)	2.76 (2.07-3.68)	30.0	2.28 (1.74-2.99)	2.34 (1.77-3.10)
>89	261(9.0)	33.1	3.09 (2.28-4.20)	2.92 (2.13-4.02)	33.7	2.82 (2.10-3.78)	3.00 (2.21-4.07)
Missing	202(7.0)	7.2	0.42 (0.22-0.81)	0.46 (0.24-0.90)	12.0	0.78 (0.46-1.33)	0.80 (0.47-1.38)
**Bilirubin**							
<= 0.4	739(25.4)	19.0	1	1	19.6	1	1
>0.4 to <= 0.6	768(26.4)	19.6	0.99 (0.78-1.25)	0.95 (0.74-1.20)	20.5	0.96 (0.76-1.21)	0.95 (0.76-1.21)
>0.6 to 0.9	675(23.2)	26.6	1.38 (1.09-1.75)	1.32 (1.04-1.68)	22.5	1.16 (0.92-1.47)	1.18 (0.92-1.50)

© 2020 Ioannou GN et al. *JAMA Network Open.*

>0.9 to 1.2	290(10.0)	20.9	1.12 (0.81-1.53)	1.11 (0.80-1.53)	23.2	1.16 (0.86-1.57)	1.19 (0.87-1.62)
> 1.2	258(8.9)	27.4	1.46 (1.08-1.98)	1.41 (1.03-1.93)	28.1	1.35 (1.00-1.82)	1.31 (0.96-1.80)
Missing	175(6.0)	6.3	0.21 (0.10-0.46)	0.25 (0.12-0.54)	12.0	0.47 (0.26-0.84)	0.49 (0.27-0.87)
**Creatinine**							
<=0.98	697(24.0)	13.2	1	1	13.6	1	1
>0.98 to 1.24	686(23.6)	18.4	1.43 (1.08-1.88)	1.35 (1.01-1.79)	14.9	1.19 (0.89-1.58)	1.22 (0.91-1.64)
>1.24 to 1.82	689(23.7)	23.0	1.79 (1.37-2.34)	1.75 (1.32-2.32)	23.9	1.72 (1.32-2.24)	1.87 (1.42-2.47)
>1.82 to 3.8	414(14.3)	36.8	2.90 (2.21-3.81)	3.24 (2.38-4.41)	36.3	2.62 (2.00-3.43)	3.05 (2.26-4.11)
>3.80	275(9.5)	27.3	2.07 (1.51-2.84)	3.30 (2.25-4.84)	30.2	2.31 (1.70-3.14)	3.79 (2.62-5.48)
Missing	144(5.0)	8.5	0.27 (0.09-0.85)	0.39 (0.13-1.23)	14.3	1.33 (0.59-3.01)	1.57 (0.70-3.52)
**Platelet count**							
>247	701(24.1)	19.6	1	1	19.2	1	1
>191 to 247	697(24.0)	19.9	1.00 (0.78-1.27)	0.94 (0.73-1.20)	16.8	0.85 (0.66-1.09)	0.82 (0.64-1.06)
>148 to 191	694(23.9)	22.7	1.18 (0.93-1.49)	1.09 (0.85-1.38)	22.8	1.17 (0.93-1.48)	1.12 (0.88-1.42)
>116 to 148	433(14.9)	23.1	1.14 (0.87-1.48)	1.04 (0.79-1.37)	24.5	1.17 (0.90-1.51)	1.13 (0.86-1.47)
<=116	283(9.7)	25.3	1.36 (1.01-1.83)	1.34 (0.98-1.83)	30.1	1.43 (1.08-1.88)	1.31 (0.97-1.75)
Missing	97(3.3)	10.0	0.10 (0.01-0.75)	0.14 (0.02-1.03)	14.3	1.11 (0.45-2.76)	1.16 (0.45-2.95)
**Hemoglobin**							
> 14.6	694(23.9)	23.9	1	1	19.8	1	1
>13.3 to 14.6	679(23.4)	21.3	0.80 (0.64-1.01)	0.83 (0.66-1.05)	17.4	0.76 (0.59-0.98)	0.79 (0.61-1.02)
>11.7 to 13.3	733(25.2)	20.7	0.80 (0.64-1.01)	0.82 (0.65-1.04)	22.1	0.81 (0.64-1.03)	0.83 (0.65-1.05)
>10.1 to 11.6	429(14.8)	19.3	0.73 (0.56-0.96)	0.76 (0.57-1.01)	24.3	0.81 (0.62-1.05)	0.78 (0.59-1.02)
<= 10.0	295(10.2)	22.6	0.83 (0.61-1.12)	0.93 (0.67-1.30)	28.0	1.01 (0.76-1.34)	0.95 (0.70-1.29)
Missing	75(2.6)	1.3	0.08 (0.01-0.56)	0.11 (0.02-0.82)	13.1	0.91 (0.37-2.25)	0.97 (0.38-2.46)
**INR**							
<= 1.04	536(18.5)	24.1	1	1	17.4	1	1
>1.04 to 1.12	535(18.4)	20.2	0.79 (0.61-1.03)	0.73 (0.55-0.96)	21.7	0.96 (0.73-1.27)	0.91 (0.69-1.21)
>1.12 to 1.29	524(18.0)	27.8	1.14 (0.89-1.48)	1.10 (0.85-1.43)	25.7	1.13 (0.86-1.49)	1.08 (0.81-1.43)
>1.29 to 1.52	318(10.9)	29.2	1.14 (0.85-1.52)	0.98 (0.73-1.33)	24.6	1.02 (0.74-1.39)	0.86 (0.62-1.19)

© 2020 Ioannou GN et al. *JAMA Network Open.*

> 1.52	210(7.2)	22.0	0.77 (0.54-1.10)	0.67 (0.46-0.97)	32.4	1.23 (0.89-1.70)	1.03 (0.73-1.44)
Missing	782(26.9)	11.9	0.36 (0.27-0.49)	0.36 (0.27-0.49)	16.1	0.77 (0.58-1.04)	0.75 (0.55-1.00)
**White Blood Cell Count**							
<= 4.77	712(24.5)	16.3	1	1	17.2	1	1
>4.77 to 6.20	716(24.6)	21.1	1.35 (1.05-1.73)	1.36 (1.06-1.76)	19.9	1.15 (0.90-1.48)	1.17 (0.91-1.50)
>6.20 to 8.30	707(24.3)	21.2	1.37 (1.07-1.75)	1.41 (1.09-1.81)	22.0	1.20 (0.94-1.53)	1.23 (0.96-1.57)
>8.30 to 11.22	424(14.6)	24.6	1.61 (1.22-2.11)	1.74 (1.32-2.30)	22.6	1.25 (0.95-1.64)	1.28 (0.97-1.69)
>11.22	284(9.8)	30.2	2.02 (1.52-2.70)	2.34 (1.74-3.14)	32.2	2.04 (1.54-2.70)	2.16 (1.62-2.87)
Missing	62(2.1)	11.7	N/A	N/A	16.4	2.17 (0.84-5.56)	2.62 (0.99-6.91)
**Neutrophil Count**							
<=3.18	695(23.9)	16.6	1	1	17.6	1	1
>3.18 to 4.5	702(24.2)	18.2	1.06 (0.82-1.37)	1.04 (0.80-1.35)	17.9	0.99 (0.76-1.28)	1.00 (0.77-1.30)
>4.50 to 6.61	687(23.6)	24.3	1.53 (1.20-1.96)	1.51 (1.18-1.93)	23.6	1.25 (0.99-1.60)	1.27 (0.99-1.62)
>6.61 to 10.14	417(14.4)	24.1	1.42 (1.08-1.87)	1.50 (1.13-1.98)	23.9	1.27 (0.97-1.66)	1.29 (0.98-1.70)
>10.14	277(9.5)	29.1	2.25 (1.64-3.11)	2.65 (1.90-3.69)	29.7	2.01 (1.47-2.74)	2.03 (1.47-2.80)
Missing	127(4.4)	18.5	1.05 (0.61-1.80)	1.18 (0.68-2.04)	20.6	1.69 (1.05-2.74)	1.75 (1.07-2.86)
**Lymphocyte Count**							
> 1.4	663(22.8)	13.3	1	1	12.2	1	1
<0.99 to 1.4	693(23.9)	21.8	1.77 (1.33-2.36)	1.67 (1.25-2.24)	19.2	1.48 (1.10-1.99)	1.44 (1.07-1.95)
<0.70 to 0.99	596(20.5)	22.9	1.87 (1.39-2.51)	1.74 (1.29-2.35)	22.6	1.67 (1.24-2.25)	1.72 (1.27-2.33)
<0.50 to 0.70	404(13.9)	25.4	2.01 (1.47-2.74)	1.95 (1.42-2.67)	27.8	2.14 (1.57-2.91)	2.14 (1.56-2.93)
<=0.50	374(12.9)	27.7	2.18 (1.59-2.98)	1.98 (1.44-2.73)	30.9	2.17 (1.59-2.94)	2.00 (1.46-2.74)
Missing	175(6.0)	19.4	1.33 (0.78-2.26)	1.41 (0.82-2.43)	23.6	2.69 (1.67-4.32)	2.69 (1.66-4.35)
**Neutrophil to Lymphocyte Ratio**							
<= 2.71	700(24.1)	12.6	1	1	12.5	1	1
>2.71 to 4.56	699(24.1)	19.0	1.59 (1.20-2.11)	1.50 (1.13-2.00)	18.9	1.53 (1.16-2.03)	1.48 (1.11-1.96)
>4.56 to 7.71	695(23.9)	23.2	1.83 (1.39-2.40)	1.71 (1.30-2.27)	23.4	1.71 (1.30-2.24)	1.71 (1.29-2.25)

© 2020 Ioannou GN et al. *JAMA Network Open.*

>7.71 to 12.70	422(14.5)	31.4	2.72 (2.04-3.62)	2.69 (2.01-3.61)	26.7	1.88 (1.40-2.52)	1.83 (1.36-2.46)
>12.70	280(9.6)	31.6	2.88 (2.10-3.94)	2.84 (2.06-3.92)	38.6	3.00 (2.23-4.05)	2.88 (2.12-3.91)
Missing	109(3.8)	12.2	0.45 (0.18-1.14)	0.45 (0.18-1.14)	13.9	1.22 (0.61-2.43)	1.20 (0.59-2.45)

* Adjusted for all the sociodemographic characteristics, comorbid conditions and symptoms listed in Tables 1-3 and stratified by station

© 2020 Ioannou GN et al. *JAMA Network Open.*
